# Editorial: Women in nutrition and food science technology

**DOI:** 10.3389/fnut.2022.1100903

**Published:** 2022-12-13

**Authors:** Elena Ibañez, Lidia Montero, Andrea del Pilar Sánchez-Camargo

**Affiliations:** ^1^Foodomics Laboratory, Institute of Food Science Research (CIAL-CSIC), Madrid, Spain; ^2^Applied Analytical Chemistry, University of Duisburg-Essen, Essen, Germany; ^3^Grupo de Diseño de Productos y Procesos (GDPP), Department of Chemical and Food Engineering, Faculty of Engineering, Universidad de Los Andes, Bogotá, Colombia

**Keywords:** gender equality, food science, sustainability, SDGs, food and health

It is interesting to see how UNESCO treats gender equality; in fact, on UNESCO's web page (https://www.unesco.org/en/gender-equality) the message is straightforward: “UNESCO believes that all forms of discrimination based on gender are violations of human rights, as well as a significant barrier to the achievement of the 2030 Agenda for Sustainable Development and its 17 Sustainable Development Goals. Our message is clear: women and men must enjoy equal opportunities, choices, capabilities, power and knowledge as equal citizens. Equipping girls and boys, women and men with the knowledge, values, attitudes and skills to tackle gender disparities is a precondition to building a sustainable future for all.” At present, <30% of researchers worldwide are women. Long-standing biases and gender stereotypes are discouraging girls and women away from science-related fields, and STEM (Science, Technology, Engineering, and Mathematics) research in particular. This shortage of women in STEM education does not come from their lack of abilities, which are, at least, equal to men's, but from the difficulties experienced (or expected) in their long-term careers. As mentioned in a post on 25 February, 2022 by Frontiers Communications in Frontiers Announcements (https://blog.frontiersin.org/2022/02/25/elena-ibanez-from-chemical-engineering-to-food-science-and-technology/), when asked about how to encourage women and girls into the scientific workforce: “the scientific community has to focus on promoting science, and not just to men. There needs to be more investment in its promotion to women, and from an early age. There needs to be constant encouragement and also continuous support even after university.” This is what we believe it can be done in order to empower all women and girls; promotion of gender equality can break stereotypes and change traditional mindsets and is essential to ensure sustainable development as highlighted by UNESCO. Therefore, all initiatives in this sense are very welcome. One of the newest is a survey promoted by the European Commission “Toward a manifesto for gender-inclusive STE(A)M education and careers” (17 October 2022) which tries to shape a roadmap of activities focused on strengthening women's and girls' participation in STEM studies and careers. Numbers are clear and we should try to balance them “Women make up 52% of the European population and make up the majority of tertiary graduates in the EU, yet only account for 2 out of 5 scientists and engineers. The gender gap widens as seniority levels increase, with women representing only 17.9% of full professors in engineering and technology fields… as highlighted in the latest She Figures 2021 Report” (1).

Likewise, for the Latin American and Caribbean region, the proportion of female researchers working in the field of engineering and technology in the region is much lower than that of men. In 2017, of the total number of researchers in engineering and technology, only 36% were women in Uruguay; 26% in Colombia; 24% in Costa Rica; 17% in El Salvador; in Honduras, 21.5%; and in Bolivia and Peru around 19% (2). The Organization of Ibero-American States for Education, Science, and Culture (OEI) also detected horizontal segregation in terms of scientific publications: women only publish 38% of the articles in physical and chemical sciences and 30% of those in engineering (2).

Bearing this in mind, in this Research Topic on *Women in Nutrition and Food Science Technology* we want to recognize and promote the work of our women scientists and colleagues working in this complex field that includes the search for an efficient and sustainable food production system and food security together with food safety and quality and aspects related to the binomial food and health and food bioactivity, always keeping in mind the achievement of the 17 Sustainable Development Goals.

This Research Topic presents a collection of manuscripts authored by women focusing on different aspects of food science technology. Very hot topics are reviewed in the three review papers published. For instance, Abril et al. addressed the use of edible insects as a sustainable food alternative in Latin America and how they can contribute to food security. Insects are nowadays seen as a food alternative not only for their easy and sustainable production but also for their high protein content and the lowest contribution to greenhouse gases compared to traditional animal-based foods. In this work, authors identify the edible insect alternatives that can be produced in Latin America and how they provide environmental, social, and nutritional benefits. Aspects such as nutritional quality, production systems, and insect-derived products are discussed, together with the current food products that are (or have the potential to) be commercialized. Another very interesting aspect in the field of food science and technology is the use of agricultural by-products and food wastes as a source of bioactive compounds that can help improve health; in this area, Andrade et al. present an interesting mini-review of the possibilities offered by coffee silverskin constituents in the prevention or co-treatment of metabolic syndrome. Other than describing the nutritional and chemical composition of this coffee by-product, the authors provide updated information on its effects on several pathways involved in the pathogenesis of metabolic syndrome. This information can help valorize coffee silverskin, which, in turn, can benefit the circular bioeconomy model. Other by-products from tiger nut (*Cyperus esculentus L*.) were studied by Pelegrín et al. in order to extract bioactive compounds and to valorize their use. A new and more environmentally friendly technique, microwave-assisted extraction (MAE), was used for extracting high-added value compounds from tiger nuts. Both, the use of food by-products and green extraction processes are in line with the circular bioeconomy, as mentioned above. The approach consisted of the study of biomass in terms of elemental composition in order to determine the most valuable fractions that could be employed for different purposes. MAE was applied to recover the polyphenolic fraction, with important antioxidant capacities, while the rest of the by-products generated during horchata preparation (the beverage obtained from tiger nuts) could have different fates other than disposal, which were also mentioned in this original research manuscript.

One very popular topic that has also been addressed in this Research Topic (Molina and Benedé) is the possible health risks associated with exposure to micro and nanoplastics in foods. Although some effects have been already identified, others have not been studied yet, such as food allergies. Plastic contamination of food and beverages has been identified as an emerging risk by the EU since they can enter the food chain through different routes, and therefore have an important impact on human health. In this mini-review, authors highlight the importance of risk assessment associated with the different potentially hazardous effects of micro- and nanoplastics can exert on the human body and focus on the nanoplastics as food allergens, which is a largely unknown topic. Moreover, they identified the gaps in the knowledge and scientific uncertainties that preclude obtaining conclusive health risk assessment of micro and nanoplastics exposure through food.

Food quality has been assessed in black walnut by Antora et al., the authors studied the metabolic profile of different black walnut cultivars in terms of vitamins, minerals, and amino acids that could be lately associated with their potential health benefits and application in the food industry. Moreover, Adainoo et al. evaluated the possibility of preserving the quality of pawpaw after freezing as a way to favor its processing and commercialization, reducing losses of this fruit. Eight cultivars were selected and a physical evaluation of the fruits was carried out; aspects considered were weight and size, color, shape, volume, density, juice content, pH, total soluble solids, thermophysical properties, and microstructure.

The effect of food/supplements intake on health has been addressed in a cross-sectional study by Andrade et al., the authors studied the relationship between vitamin D intake from food/supplements and factors that may be associated with self-reported vitamin D deficiency among US adults. The study draws some interesting conclusions such as that through self-reporting, the prevalence of vitamin D deficiency was nearly 39%; that foods naturally rich in vitamin D were rarely/never consumed, and that although intake of vitamin D supplements occurred frequently, associations could be found with certain chronic conditions/diseases and vitamin D deficiency. The authors suggested appropriate nutrition education programs to help individuals to consume and prepare foods naturally high in vitamin D to reduce the prevalence of vitamin D deficiency.

Another topic treated in this RT is the use of freshwater macrophytes to feed animal food sources such as fish, poultry, and livestock (Kumar et al.). The authors evaluate the nutritional value of different freshwater cultured macrophytes in terms of proteins, lipids, and minerals. The study revealed that these aquatic plants were rich in polyunsaturated fatty acids (PUFAs) and minerals; while shedding some light on the most appropriate cultured macrophyte to be used as a feed ingredient.

Summarizing, this Research Topic presented interesting results in different aspects related to food science technology, in very hot topics, all of them led by women, showing their excellence and leadership. We are grateful to all of them, their research teams, and their colleagues for their excellent contributions in this complex and exciting area. We hope that their contribution to this special *Women in Nutrition and Food Science Technology* issue serves as an example of the excellent research potential of women in Science.

## Author contributions

EI supervised and wrote the editorial article. LM and AS-C reviewed the final version of the editorial article. All authors approved the final version of the manuscript for publication.
